# Drug-Initiated Synthesis and Processing of Mupirocin–PLGA Conjugates for Biodegradable Antibacterial Scaffolds

**DOI:** 10.1021/acsomega.6c06646

**Published:** 2026-10-05

**Authors:** Tomasz Urbaniak, Zuzanna Podgórniak, Adam Junka, Jolanta Harasiuk, Witold Musiał

**Affiliations:** † Department of Physical Chemistry and Biophysics, Faculty of Pharmacy, 49550 Wroclaw Medical University, Borowska 211, Wrocław 50-556, Poland; ‡ Platform for Unique Model Applications (PUMA), Division of Translational Technologies, Faculty of Pharmacy, 49550 Wroclaw Medical University, Borowska 211, Wrocław 50-556, Poland; § College of Life Sciences and Medicine, Zhejiang Sci-Tech University, 928 Second Avenue, Xiasha Higher Education Zone, Hangzhou, Zhejiang 310018, P.R.china; ∥ Department of Molecular and Cellular Biology, Faculty of Pharmacy, 49550 Wroclaw Medical University, Borowska 211a, Wroclaw 50-556, Poland

## Abstract

Mupirocin–poly­(lactic-*co*-glycolic acid) (Mup-PLGA) conjugates were developed via drug-initiated ring-opening polymerization to create biodegradable scaffolds with local antibacterial activity. Conjugate composition and molecular weight were controlled to tune degradation behavior, and materials were processed into porous solvent-cast scaffolds and electrospun mats. Degradation studies showed that molecular weight, copolymer ratio, and plasticizer content governed structural evolution, while the processing method had a limited effect. No measurable release of free mupirocin was detected during degradation. Despite this, antibacterial activity was observed under direct-contact conditions and correlated with conjugate composition, reflecting mupirocin content along polymer chains. Electrospun scaffolds exhibited limited diffusible activity, attributed to higher surface area. Overall, the materials did not behave as classical release systems but showed surface-mediated antibacterial effects instead. These findings position Mup-PLGA conjugates as a platform for wound dressings combining tunable degradation with local antimicrobial functionality, potentially reducing exposure to subinhibitory free antibiotic concentrations. Extract-based MTT testing showed mean cell viability above the 70% criterion for both electrospun mats and the nonplasticized SC7 scaffold, whereas TEC-containing solvent-cast scaffolds were cytotoxic.

## Graphical abstract

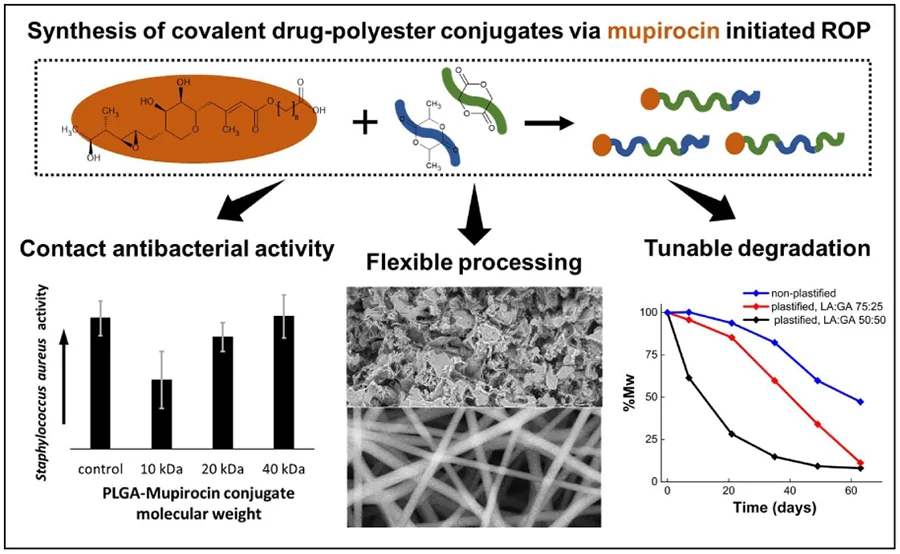

## Introduction

Nonhealing wounds, such as diabetic foot ulcers, venous leg ulcers, pressure sores, and burns are a major healthcare challenge of profound individual and socio-economic impact. Affecting an estimated 1–2% of the population in developed countries, they are particularly prevalent among elderly and diabetic patients, leading to long-term morbidity, reduced quality of life, and, in severe cases, limb amputation or death. In the U.S. alone, over 6.5 million individuals suffer from nonhealing wounds, with annual treatment costs exceeding $25 billion. This economic burden is expected to rise due to aging populations and increasing rates of diabetes and obesity.^1^

One of the key factors underlying impaired wound healing is persistent bacterial infection. Opportunistic pathogens, including *Staphylococcus aureus* and *Pseudomonas aeruginosa*, frequently colonize the wound bed and form biofilms that protect bacteria from host defenses and antibiotic therapy. These infections prolong inflammation, disrupt tissue regeneration, and contribute to chronicity.^2^ Current management of nonhealing wounds is based on wound bed preparation strategies, including debridement, exudate control, and the use of advanced dressings, with antimicrobial therapy reserved for clinically infected wounds. Nevertheless, effective local infection control remains difficult: systemic antibiotics may not achieve sufficient concentrations in poorly vascularized tissue, while topical antimicrobials often require repeated application, leading to fluctuating local drug levels and potential exposure of bacteria to subinhibitory concentrations, which may contribute to the development of resistance.^3^ These limitations highlight the need for materials capable of providing localized antibacterial activity while minimizing unnecessary drug exposure.

Biomaterial-based wound dressings have emerged as a promising approach to simultaneously support tissue regeneration and control infection. Modern scaffolds, including hydrogels, films, porous sponges, and electrospun nanofibers, are designed to mimic aspects of the extracellular matrix, facilitate cell infiltration, and, when required, deliver therapeutic agents in a controlled manner.^4^ Among these materials, poly­(lactic-*co*-glycolic acid) (PLGA) is widely used due to its biocompatibility and tunable degradation, which can be adjusted through copolymer composition and molecular weight to match the wound healing process.^5^ Beyond copolymer composition and molecular weight, molecular architecture and pendant-group chemistry provide additional means of controlling the properties of biodegradable polyesters. Studies on poly­(alkyl glycolide)–polyglycolide copolymers demonstrated that the nature of the alkyl substituent influenced glass-transition behavior, surface properties, and hydrolytic degradation. Greater molecular-weight reduction and mass loss were observed for a copolymer whose T_g_ was below the degradation temperature, which was attributed to increased segmental mobility and facilitated water transport through the polymer matrix.^6^ Polymer topology can also affect polyester properties. Comparisons of linear and four-arm star-shaped poly­(substituted glycolide)­s revealed differences in thermal behavior and crystallinity. Increasing the alkyl side-chain length also increased surface hydrophobicity and roughness while reducing surface free energy.^7^ Although these polymers are chemically distinct from PLGA, they illustrate how polyester architecture and side-chain structure can influence degradation-relevant physicochemical properties and interfacial behavior. Incorporation of antimicrobial functionality into such scaffolds is commonly achieved through physical drug loading, where antibacterial efficacy is primarily governed by diffusion-driven release into the surrounding medium. While effective, these systems often exhibit burst release and sustained exposure of bacteria to subinhibitory concentrations, which may contribute to resistance development. An alternative strategy are polymer–drug conjugates, in which therapeutic molecules are covalently bound to biodegradable polymers. This approach is typically explored in the context of controlled release, where drug liberation is coupled to polymer degradation or linker cleavage.^8^ However, the possibility that such systems may act without significant release, instead exerting antibacterial effects at the material interface, remains less explored, particularly for antibiotic-functionalized biodegradable polyesters.

In the case of PLGA, postsynthetic conjugation has been demonstrated for several small molecules, including anticancer agents, but examples involving antibiotics are limited.^9–14^ Extending this concept to antibacterial systems raises fundamental questions regarding the relationship between conjugate composition, degradation behavior, and mode of antibacterial action. In particular, it remains unclear whether effective antibacterial activity can be achieved in the absence of measurable drug release and how material structure influences this behavior.

Mupirocin (MUP) is a clinically relevant antibiotic that inhibits bacterial isoleucyl-tRNA synthetase and is especially effective against *S. aureus* and MRSA. Despite its efficacy, its clinical use is limited by a short half-life and the need for frequent topical application. Its hydrophobic structure and functional groups compatible with polyester chemistry make it a suitable candidate for incorporation into biodegradable polymer systems via covalent linkage.^15^ Despite its clinical effectiveness, increasing resistance to MUP has been reported, particularly in settings involving prolonged or repeated topical use.^16^ This trend raises concerns regarding the long-term utility of this antibiotic and highlights the need for strategies that preserve its effectiveness. In this context, material-based approaches that limit drug diffusion while maintaining antibacterial activity at the material interface may reduce exposure of bacteria to subinhibitory concentrations, thereby lowering selective pressure for resistance development and supporting more sustainable use of mupirocin in wound care applications.

In this work, MUP was used as an initiator in the ring-opening polymerization of lactide (LA) and glycolide (GA) to obtain Mup-PLGA conjugates with controlled composition and molecular weight. The resulting materials were processed into two scaffold types with distinct architectures: porous structures prepared by solvent casting and salt leaching, and nanofibrous mats fabricated by electrospinning. These systems were used to investigate how conjugate design and scaffold morphology influence degradation behavior and antibacterial performance. Particular emphasis was placed on distinguishing between diffusion-driven and contact-mediated antibacterial mechanisms, providing insight into the potential of nonleaching, surface-active biomaterials for wound dressing applications.

## Materials and Methods

### Materials

l-Lactide (LA, 95%) and glycolide (GA, 97%) were obtained from BLDpharm (USA), recrystallized from ethyl acetate, and stored in a desiccator prior to use. Mupirocin (946 μg/mg purity) was used as received. Sodium chloride (NaCl) was ground with a mortar and pestle and sieved to isolate particles between 53 and 106 μm. Dichloromethane (CH_2_Cl_2_), dimethylformamide (DMF), chloroform (CHL), ammonium acetate, triethyl citrate, tributyl citrate and all other reagents were of analytical grade and used without further purification. Saline (0.9% NaCl), ethanol, isopropanol, PBS (POCH, Poland, Lublin); MHA agar (Mueller–Hinton, Biomaxima, Lublin, Poland); Mupirocin ointment (Mupirox, Bausch Health Ireland); TTC (Pol-Aura, Warszawa)

### Synthesis of Mup-PLGA Conjugates

Mup-PLGA conjugates were synthesized by bulk ROP of LA and GA in the presence of MUP, using stannous octoate (Sn­(Oct)_2_) as the catalyst. Reactions were carried out in sealed three-necked glass flasks equipped with septa under a constant nitrogen atmosphere. A total of 2.0 g of monomers was used in each reaction, combined at molar ratios of 50:50, 75:25, or 35:65 (LA:GA). The molecular weight of the resulting polymers (10, 20, 40, or 80 kDa) was controlled by varying the amount of MUP, which acted as the initiator. Accordingly, the monomer-to-initiator ratio determined the targeted degree of polymerization and thus the theoretical molecular weight of the conjugates. The reaction mixtures were stirred and heated to 130 °C, after which 10 μL of Sn­(Oct)_2_ was added to initiate polymerization. The reactions were allowed to proceed for 3 h. The crude products were then dissolved in chloroform and precipitated into cold methanol. The resulting polymers were collected by vacuum filtration and dried under reduced pressure at room temperature for 48 h.

To evaluate MUP stability under conditions approximating the polymerization environment, MUP was incubated in a molten LA/GA mixture corresponding to the 75:25_10 formulation. LA (750.8 mg), GA (201.5 mg), and MUP (47.7 mg) were heated at 130 ± 1 °C for 180 min under a nitrogen atmosphere without Sn­(Oct)_2_. Aliquots were collected at 0, 15, 30, 60, 120, and 180 min and dissolved in ACN. The concentration of intact MUP was determined using the HPLC method and normalized to the value measured at 0 min.

### ^1^H Nuclear Magnetic Resonance Spectroscopy

^1^H NMR spectra were recorded on a Bruker AVANCE III 600 MHz spectrometer at 298 K. Samples (10 mg) were dissolved in 0.7 mL of CDCl_3_, and spectra were acquired using a zg30 pulse sequence with a 30° flip angle, a relaxation delay of 2 s, and 16 scans per sample. Chemical shifts (δ) were referenced to the residual CHCl_3_ peak at 7.26 ppm. Data were processed and analyzed using Bruker TopSpin software.

The LA:GA copolymer ratio was determined from the relative integrated areas of the LA methine signal at 1.60 ppm and the GA methylene signal at 4.84 ppm. Molecular weight and degree of MUP incorporation were calculated from the ratio of the LA/GA signals to the MUP vinyl proton resonance at 5.76 ppm.

### Differential Scanning Calorimetry

Thermal transitions of Mup-PLGA conjugates were measured on a DSC 214 Polyma (Netzsch, Selb, Germany). About 5 mg of sample was sealed in pierced aluminum pans and heated from −10 to 120 °C at 5 °C/min under nitrogen (250 mL/min). Two heating–cooling cycles were recorded, with the second heating run in modulated mode (±0.5 °C every 60 s). Glass transition temperatures (T_g_) were determined from the midpoint of the heat flow shift in the second heating curve using Proteus software.

### Gel Permeation Chromatography

Molecular weights (M_n_, M_w_) and polydispersity index (PDI) of the synthesized conjugates and degradation samples were determined using a Thermo Scientific Dionex UltiMate 3000 HPLC system with a RefractoMax 521 RI detector. Samples (5 mg/mL in DMF) were injected (100 μL) onto a Phenogel column (10^3^ Å, 5 μm, 300 × 7.5 mm) at 25 °C. THF was used as the mobile phase at 1.0 mL/min. Molecular weights were calculated against polystyrene standards (Supelco, USA).

### Scaffold Fabrication by Solvent Casting and Salt Leaching

Plasticizer screening was first performed using the 75:25_10 Mup-PLGA conjugate. Mixtures of 75 mg polymer, 375 mg sieved NaCl (53–106 μm), and varying concentrations of triethyl citrate (TEC) or tributyl citrate (TBC) (5, 10, or 15% w/w relative to polymer mass) were dissolved in 0.5 mL CH_2_Cl_2_, cast into Teflon molds (12.5 × 25 × 3 mm), left to dry for 2 h, and subsequently immersed in distilled water for 3 h to leach out the salt. Scaffold integrity and porosity were evaluated to identify suitable plasticizer conditions, which were then applied for the preparation of scaffolds from selected Mup-PLGA conjugates by scaling up to 450 mg polymer, 2.25 g NaCl, and 3.0 mL CH_2_Cl_2_ with 10% w/w TEC casted into Teflon mold (75 × 25 × 3 mm); scaffold SC7 (75:25_80 Mup-PLGA) was prepared without plasticizer.

### Scaffold Fabrication by Electrospinning

Electrospun mats were produced from Mup-PLGA conjugates. Initial screening was performed using three polymers (75:25_20, 75:25_40, 75:25_80) in different solvent systems (CHL:DMF and THF:DMF at varying ratios) and voltages from 8 kV upward in 2 kV increments (see Table 1). Mats selected for degradation and microbiological studies (ES1 and ES2) were prepared from 75:25_40 (40% w/v) and 75:25_80 (35% w/v) in CHL:DMF 7:3 v/v at 12 kV. Solutions were electrospun using an E-Fiber EF050 electrospinning system (SKE, Bollate, Italy) equipped with a 17 G needle at a flow rate of 1.0 mL/h and a 10 cm needle-to-collector distance onto a flat collector plate under ambient conditions (20–22 °C, 40–50% RH).

**Table 1:** Summary of Key Structural and Physicochemical Parameters of Synthesized Conjugates

Synthesis	*M*_w_ ^a^ [kDa]	PDI^a^	M_n_/NMR^b^ [kDa]	LA:GA ratio (product)^b^	MUP content^b^	Yield [%]	T_g_ [°C]
75:25_10	11.87	1.94	8.86	75:25	5.7%	72%	30.4
75:25_20	18.4	2.23	17.34	75:25	2.9%	74%	30.6
75:25_40	31.1	2.49	33.46	75:25	1.5%	84%	31.1
75:25_80	41.9	2.12	74.29	74:26	0.7%	88%	35.9
50:50_10	9.0	1.95	8.40	52:48	6.0%	83%	21.6
50:50_20	13.1	2.17	14.79	49:51	3.4%	73%	28.8
50:50_40	10.2	1.97	35.93	49:51	1.4%	68%	33.7
50:50_80	16.4	2.57	75.15	51:49	0.7%	74%	34.3
35:65_10	13.6	1.81	10.65	39:61	4.7%	89%	39.1
35:65_20	18.9	2.22	19.90	41:59	2.5%	89%	40.3

a Determined via GPC.

b Determined via ^1^H NMR.

### Scanning Electron Microscopy

Scaffold and electrospun mat morphology was examined using a Phenom ProX G6 SEM in low-vacuum mode without sputter coating. Samples were mounted on aluminum stubs and imaged at accelerating voltages of 10–15 kV, depending on the sample. Magnifications varied to capture relevant features, with scale bars included in all images.

### In Vitro Degradation and Drug Release Analysis

Scaffold samples (100 mg) were incubated in 1.5 mL phosphate-buffered saline (PBS, pH 7.4) in 1.5 mL microcentrifuge tubes sealed with Parafilm. Samples were shaken at 200 rpm on an orbital shaker at 32 °C. The temperature of 32 °C was selected to approximate the local thermal conditions of the wound bed rather than core body temperature. Chronic wound-bed temperatures have been reported to generally fall within the range of 30.2–33.0 °C,^17^ supporting the relevance of this temperature for evaluating scaffolds intended for topical wound applications. For drug release studies, 0.2 mL samples were collected at ten selected time points over 9 weeks, with the medium replenished by fresh PBS at each collection.

Chromatograms of the release media were recorded immediately after collection using HPLC on a HITACHI Primaide system with UV–vis detection at 228 nm. Samples (20 μL) were injected onto a Lichrospher Select B C8 column (250 × 4 mm, 5 μm). The mobile phase was 0.05 M ammonium acetate:acetonitrile (72.5:27.5 v/v) at a flow rate of 1.0 mL/min.^18^ Calibration was performed using a standard MUP curve (R^2^ = 0.999).

For polymer degradation studies, 10 mg scaffold samples were incubated under the same conditions in 0.5 mL PBS and collected at five selected time points for GPC analysis. For SEM analysis, two circular samples of each scaffold type (diameter 5.5 mm) were incubated in 0.5 mL PBS and collected at three time points.

### Microbiological Studies

#### Confirmation of Susceptibility of *Staphylococcus aureus* to MUP

The susceptibility of *S. aureus* ATCC 6538 to MUP was confirmed using a standard well-diffusion assay. Bacterial suspensions were prepared in sterile saline (0.5 McFarland, ≈1.5 × 10^8^ CFU/mL) and uniformly spread onto Mueller–Hinton agar (MHA) plates. After drying, a central well (3 mm diameter) was cut into the agar using cork-borer and filled with 500 mg of a reference MUP were incubated aerobically at 37 °C for 24 h, and inhibition zones were measured.

#### Antimicrobial Activity of Scaffold Samples (Disk Diffusion Assay)

Bacterial lawns of *S. aureus* ATCC 6538 were prepared on MHA plates as described above. Circular scaffold disks (5.5 mm diameter) from SC1–SC7 and ES1–ES2 were placed on the agar surface. Blank disks without MUP served as negative controls, and disks containing 1.5 μL DMF were included to account for residual solvent from electrospinning. Plates were incubated at 37 °C for 24 h, and growth inhibition zones were measured.

#### Bacterial Adherence to Scaffold Disks (Contact TTC Assay)

Direct-contact antibacterial activity was evaluated using a TTC-based metabolic assay. Circular disks (5.5 mm diameter) were placed on MHA plates, and 10 μL of *S. aureus* suspension (0.5 McFarland) was applied onto each disk. After 3 h incubation at 37 °C, 5 μL of 1% (w/v) TTC solution was added, followed by an additional 3 h incubation. Nonadhered cells were removed, and disks were transferred to 96-well plates containing 200 μL extraction buffer (ethanol:isopropanol:1 M HCl, 95:4:1 v/v/v). After 10 min agitation, absorbance was measured at 490 nm. Samples without MUP were defined as 100% metabolic activity.

#### Evaluation of Antibacterial Activity of Scaffold Release Media

Electrospun samples (ES1 and ES2; 15 × 15 mm) were incubated individually in 1.5 mL PBS (pH 7.4) at 32 °C under agitation. Separate specimens were incubated for 1, 2, or 3 weeks. At each end point, the entire release medium was collected and analyzed without dilution. Antibacterial activity was assessed using a broth microdilution method in sterile, flat-bottom 96-well plates. Bacterial suspensions were prepared as described above and diluted in Mueller–Hinton broth (MHB) to obtain a final inoculum of approximately 5 × 10^5^ CFU/mL. 2-fold serial dilutions of release media were prepared directly in the microplate. Growth and sterility controls were included, and plates were incubated at 37 °C for 18–24 h. Bacterial growth was evaluated visually and quantified using TTC reduction (5 μL of 1% TTC, 2 h incubation, absorbance at 490 nm; Tecan Spark GO). In parallel, the minimum inhibitory concentration (MIC) of MUP was determined under identical conditions using 2-fold serial dilutions of the reference compound. The MIC was defined as the lowest concentration completely inhibiting visible growth.

#### In Vitro Cytocompatibility Assessment

The cytocompatibility of Mup-PLGA scaffolds was evaluated using an extract-based MTT assay with HaCaT human keratinocytes, with reference to ISO 10993-5 and ISO 10993-12.^19,20^ Scaffold samples were incubated in complete high-glucose DMEM containing l-glutamine and sodium pyruvate (Biowest, L0104), supplemented with 10% fetal bovine serum (EURx, E5053-02) and 1% Antibiotic-Antimycotic solution (Gibco, 15240-062), at a ratio of 0.1 g of sample per 1 mL of medium for 24 h at 37 °C under static conditions. The extracts were filtered through sterile 0.2 μm syringe filters and used without dilution. HaCaT cells (Cytion, 300493) were maintained in the same complete medium at 37 °C in a humidified atmosphere containing 5% CO_2_. Cells were seeded in 96-well plates at 8,000 cells per well in 200 μL of medium and cultured for 24 h before exposure. The culture medium was then replaced with 125 μL of undiluted scaffold extract, and the cells were incubated for 24 h. Subsequently, 12.5 μL of MTT solution (5 mg/mL; PanReac AppliChem, A2231.0010) was added to each well and incubated for 2 h. The medium was then removed, and the resulting formazan crystals were dissolved in 50 μL of dimethyl sulfoxide by incubation with gentle agitation for 20 min. Absorbance was measured at 560 nm using a GloMax Discover Microplate Reader (Promega, USA). Cells exposed to complete medium processed under the same conditions as the extracts served as the viability control, while wells containing medium without cells were used for background correction. Each condition was evaluated in five technical replicate wells. Cell viability was calculated from blank-corrected absorbance values and expressed as a percentage of the untreated control. Values are presented as mean ± SD. Cell viability below 70% of the untreated control was considered indicative of a cytotoxic effect according to ISO 10993-5.

## Results and Discussion

### Mup-PLGA Characterization

The polymerization temperature of 130 °C was selected taking into account previously reported thermogravimetric data for mupirocin. Ridichie et al. reported that the onset of MUP mass loss occurred at 148 °C at a heating rate of 2 °C min–1 and shifted to 164–173 °C, at higher heating rates.^21^ Although these results indicate that 130 °C is below the reported onset of substantial thermogravimetric decomposition, dynamic TGA measurements do not establish molecular stability during prolonged isothermal exposure. Therefore, MUP stability was additionally evaluated by incubation in the molten LA/GA reaction mixture at 130 °C for 180 min under nitrogen, without Sn­(Oct)_2_. HPLC analysis showed a gradual reduction in apparent intact-MUP recovery, reaching 75.6% of the initial value after 180 min (Figure S1). The results indicate partial degradation or transformation of MUP during prolonged heating and do not support complete thermal stability under these conditions. However, this experiment does not fully reproduce the polymerization process because Sn­(Oct)_2_ was omitted to prevent simultaneous MUP-initiated polymerization. The presence of the catalyst and the progressively forming polymer matrix may modify the stability, reaction pathway, and incorporation of MUP. The products of drug-initiated ROP were characterized using ^1^H NMR and GPC to confirm their chemical structure and the presence of covalently bound MUP as a terminal group on the PLGA chains. According to the established ROP mechanism, hydroxyl-containing initiators—such as MUP—interact with monomers via a catalyst-mediated coordination–insertion mechanism. This initiates chain propagation and determines the polymer architecture.^22^ Consequently, the molar ratio of MUP to monomer units in the reaction mixture directly influences the final polymer chain length. Consistent with this theoretical framework, the molecular weight of the resulting polymers correlated with the employed feed ratios of monomers and initiator (Table 1). Synthesis codes follow the format LA:GA_M_w_, where LA:GA indicates the monomer feed ratio, and M_w_ denotes the targeted molecular weight (kDa).

The agreement between theoretical and GPC-derived molecular weights, particularly in low-molecular-weight samples, supports the successful covalent incorporation of MUP into the PLGA chains. In addition, ^1^H NMR analysis yielded molecular weight values that closely matched the theoretical ones across nearly all samples, further confirming the efficiency of the initiation process. By contrast, the deviations observed in GPC at higher monomer-to-initiator ratios can be attributed to intrinsic limitations of drug-initiated ROP systems, as the rise in viscosity during bulk polymerization restricts mass transfer and catalyst mobility, leading to heterogeneous propagation, premature chain termination, and broader molecular weight distributions.^23,24^ Collectively, these effects may account for the observed discrepancies from theoretical molecular weights in higher molecular weight formulations. The moderately broad polydispersity indices (PDI), ranging from 1.8 to 2.6 across all Mup-PLGA conjugates, further suggest the occurrence of side reactions such as transesterification or uncontrolled initiation by impurities. The T_g_ determined for the obtained conjugates increased with glycolide content, reaching the highest values for GA-rich 35:65 compositions. In contrast, more LA-rich systems exhibited lower T_g_ values around 30–36 °C, while 50:50 copolymers showed intermediate behavior (Table 1, Figure S2A). Within a given LA:GA ratio, an increase in molecular weight led to a modest but consistent elevation in T_g_. This effect can be attributed to the decreasing relative concentration of chain ends in higher-molecular-weight polymers, which reduces chain-end mobility and thus restricts local segmental motions.^25^ The interplay between monomer composition and molecular weight therefore defines the observed glass-transition behavior, with glycolide-rich and higher-molecular-weight conjugates exhibiting the highest T_g_ values in the temperature range relevant for scaffold performance.

The ^1^H NMR spectra of the Mup-PLGA conjugates were dominated by intense, clustered signals corresponding to LA and GA monomer units (Figure 1A).

**Figure 1:**
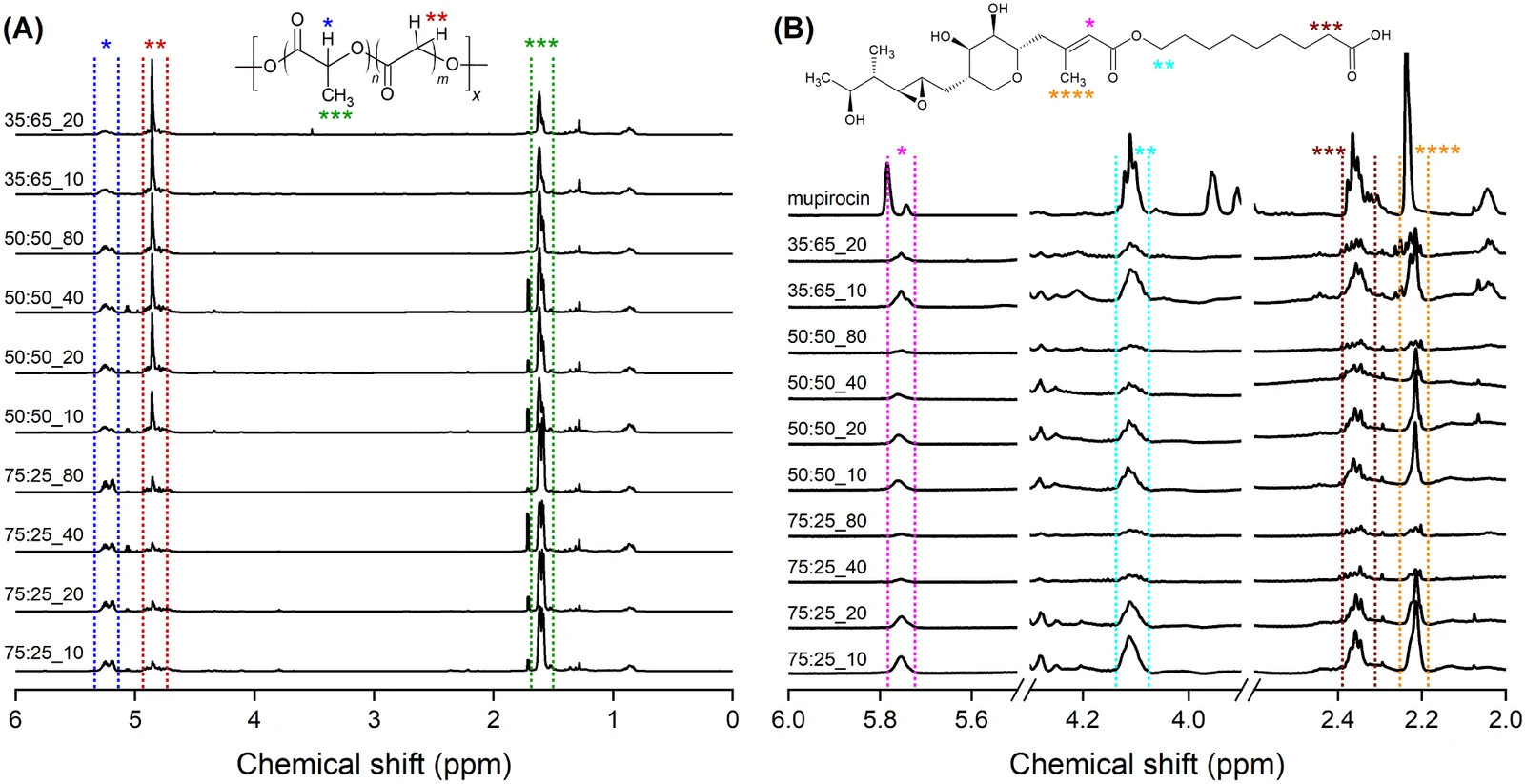
^1^H NMR spectra of Mup-PLGA conjugates. (A) Full spectra with backbone protons from LA and GA assigned to peaks, (B) spectral regions highlighting MUP-derived signals with selected protons annotated.

The ratio of monomer units in the resulting polymer chains is a key parameter determining the hydrophilicity of the biomaterial, and consequently, the degradation rate—an essential factor for biomedical applications. The monomer composition of the polymeric products, calculated from the integration of selected peaks, closely reflected the feed ratios used during synthesis (Table 1). Signals originating from MUP protons were of low intensity due to the relatively low MUP content, appearing near the baseline. Nevertheless, distinct proton signals could be assigned to the β-alkene carbon and vinylic methyl group of monic acid, as well as to protons adjacent to the carboxyl and ester groups of the hydroxynonanonic acid moiety of MUP (Figure 1B).^26^ Relative height of MUP-derived peaks decreased systematically with increasing molecular weight. Integration of selected resonances revealed a decrease in MUP content with increasing molecular weight: ∼5–6% in the lowest-M_w_ conjugates, ∼1–3% at intermediate M_w_, and below 1% in the highest-M_w_ samples (Table 1). This trend reflects the progressively smaller molar contribution of MUP to the polymer chains as chain length increases, in agreement with the designed stoichiometry.

### Scaffold Preparation via Solvent-Casting Salt Leaching (SCSL) Method

The mechanical properties of PLGA, which are critical for processing into 3D scaffolds, are primarily governed by its molecular weight, as this affects the degree of chain entanglement and intermolecular interactions.^27^ Low molecular weight PLGA (<50 kDa) tends to be brittle and fragile, whereas higher molecular weight polymers exhibit greater tensile strength and elasticity, making them more suitable for fabrication and use in biomedical devices. This is particularly important for scaffolds produced via the SCSL method, where thin pore walls, formed after leaching, are prone to fragmentation.^28^ Given the low molecular weight of the synthesized Mup-PLGA conjugates, the effect of biocompatible plasticizers—TBC and TEC—on the morphology and T_g_ of SCSL-fabricated scaffolds was investigated (Figure 2, Figure S2B).

**Figure 2:**
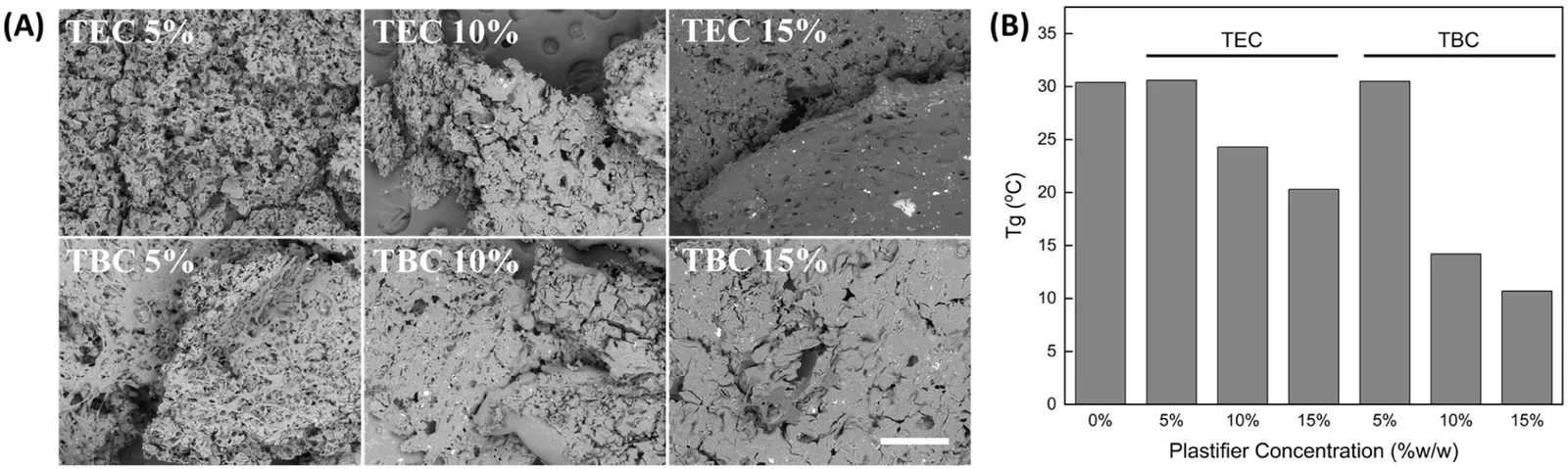
Effect of plasticizer type and concentration on the morphology and thermal properties of 75:25_10 Mup-PLGA scaffolds. (A) Representative SEM images of scaffolds with different plasticizers and concentrations, (B) glass transition temperature values for each scaffold variant.

Due to significant brittleness and poor structural integrity after drying, scaffolds prepared from the 75:25_10 Mup-PLGA conjugate were selected for plasticizer screening. The addition of 10% and 15% TBC or TEC resulted in handleable scaffolds, meaning they could be manipulated with forceps and exhibited sufficient elasticity to allow bending without breaking. DSC curves of all scaffolds prepared with different plasticizer concentrations revealed single inflection point reflecting T_g_ of investigated materials (Figure S2B). The improved mechanical performance is attributed to plasticizer-induced reduction in intermolecular forces and increased polymer chain mobility, which also impact T_g_ of blends (Figure 2A).^29^ Plasticization visibly altered the scaffold morphology, only scaffolds prepared with 5% and 10% TEC and 5% TBC displayed defined porosity, reflecting the shape of the salt crystals used as porogen. For both plasticizers, concentration dependent impact on T_g_ was observed, with more pronounced effect for TBC. These differenced are reflected in morphology of obtained structures, where material with lower softening temperature lost porosity, presumably due to collapse of thin walls composed of soft, plasticized material. Consequently, 10% TEC was selected as the formulation for processing most conjugates into scaffolds intended for degradation studies, as it provided acceptable mechanical properties while preserving porosity. Solvent-cast scaffolds (SC1–SC7) were ultimately prepared from Mup-PLGA conjugates with different copolymer ratios and molecular weights: SC1–SC3 from 50:50 conjugates (10, 20, and 40 kDa respectively) and SC4–SC7 from 75:25 conjugates (10, 20, 40, and 80 kDa, respectively). Notably, SC7 was processed without plasticizer, as its mechanical properties were acceptable without plasticization, likely due to the highest molecular weight among the tested conjugates. Other synthesized conjugates did not exhibit sufficient solubility to enable processing by the SCSL method.

### Electrospinning of Mup-PLGA

Electrospinning was attempted for three Mup-PLGA conjugates (75:25 LA:GA) with molecular weights of 20, 40, and 80 kDa. Other synthesized variants were not evaluated due to insufficient solubility in the investigated solvent systems, or, in the case of 75:25_10, due to the low molecular weight. The resulting fiber morphology strongly depended on both polymer molecular weight and concentration (Table 2).

**Table 2:** Summary of Electrospinning Screening for Mup-PLGA Conjugates, Including Scaffold Morphology Observed by SEM

Synthesis product	Concentration	Solvent system	Voltage [kV]	Resulting morphology
75:25_80	30%	CHL:DMF 6:4	8–12	beading
		THF:DMF 6:4	8–12	beading
	35%	CHL:DMF 6:4	8–12	beading
			14	sparse beading
		THF:DMF 6:4	8–14	beading
		CHL:DMF 7:3	8–14	fibers
		THF:DMF 7:3	8–14	irregular fibers
75:25_40	35%	CHL:DMF 7:3	8–16	beading
	35%	THF:DMF 7:3	8–16	beading
	40%	CHL:DMF 7:3	8–16	irregular fibers
	40%	THF:DMF 7:3	8–16	beading
75:25_20	40%	CHL:DMF 7:3	8–10	coalesced particles
			12–14	microparticles
	40%	THF:DMF 7:3	8–10	coalesced particles
			12–14	microparticles

The 20 kDa conjugate did not form fibers at any tested concentration. Electrospraying dominated, producing coalesced droplets at low voltages (8–10 kV) and discrete microparticles at higher voltages (12–14 kV) (Figure S5). This reflects insufficient chain entanglement, consistent with prior observations for low-M_w_ PLGA below the critical entanglement concentration.^30^ The 40 kDa conjugate displayed transitional behavior. At 35% (w/v), only beaded structures were observed across all voltages (Figure S4). Increasing the concentration to 40% (w/v) enabled irregular fiber formation in chloroform-rich systems, though beads remained present. This indicates that the polymer concentration was near the threshold required for stable jet elongation, in agreement with literature reports showing that higher polymer loading reduces bead formation but does not guarantee uniform fibers.^30,31^ The 80 kDa conjugate exhibited improved fiber-forming ability compared to lower-molecular-weight analogs. At 30% (w/v), beading persisted, but at 35% (w/v) continuous fibers were obtained in chloroform-rich mixtures across 8–14 kV (Figure S3). Less favorable solvent systems yielded irregular fibers or beading, highlighting the combined influence of polymer concentration and solvent composition on jet stability. This behavior aligns with previous studies demonstrating that high-M_w_ PLGA requires sufficiently concentrated solutions and appropriate solvent volatility to produce uniform fibers.^32,33^ Solvent composition significantly affected morphology for all molecular weights. Chloroform-rich mixtures consistently promoted fiber formation, whereas THF-based systems generally produced beads or irregular fibers. These trends are consistent with reports that solvent volatility and miscibility strongly influence jet stretching and solidification.^30,33^ The presence of MUP in conjugate may slightly impact electrospinning process. Its polar moieties could affect viscosity or conductivity, potentially shifting the entanglement threshold compared with unmodified PLGA. However, the dominant determinants of morphology remained molecular weight, polymer concentration, and solvent composition, with end-group effects likely secondary. Electrospun scaffolds selected for degradation and microbiological studies ES1 and ES2 were produced from the 75:25_40 and 75:25_80 conjugates, respectively.

### Scaffold Degradation Study

PLGA undergoes hydrolytic chain scission of its ester linkages, producing progressively shorter oligomers and acidic byproducts that change mechanical and morphological properties over time.^34^ To assess molecular and morphological evolution of the Mup-PLGA scaffolds, molecular-weight changes were monitored by GPC and microstructure by SEM over a 63-day incubation period. GPC traces separate the data set into three distinct decay patterns corresponding to (i) plasticized 50:50 scaffolds (SC1–SC3), (ii) plasticized 75:25 scaffolds (SC4–SC6), and (iii) nonplasticized scaffolds (SC7 and the ES1, ES2 electrospun mats) (Figure 3).

**Figure 3:**
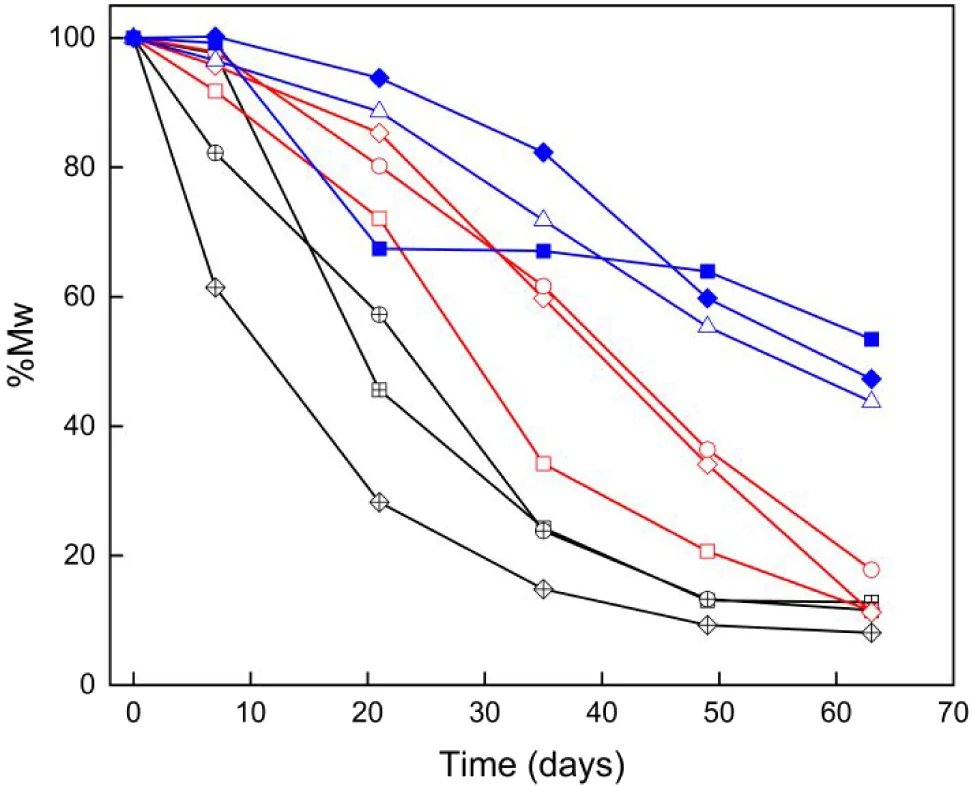
Degradation of Mup-PLGA solvent-casted scaffolds (SC1–SC7) and electrospun mats (ES1, ES2) over 63 days, shown as percent of initial molecular weight (%M_w_). Colors indicate formulation type: blue – TEC-free scaffolds/mats (SC7: empty triangle; ES1: filled square; ES2: filled rhombus), red – 75:25 scaffolds with TEC (SC4: empty circle; SC5: empty square; SC6: empty rhombus), black – 50:50 scaffolds with TEC (SC1: empty circle with cross; SC2: empty square with cross; SC3: empty rhombus with cross).

Both plasticized sets converged into the low-kDa domain: several plasticized samples reached ≲2 kDa by the end of the experiment, while others in the plasticized 75:25 set retained higher residual chain length on the order of ∼5–6 kDa (Figure S6A). By contrast, the nonplasticized materials maintained substantially higher M_w_ at the final time point, with SC7 remaining around 18 kDa and the electrospun mats in the 16–20 kDa range. Polydispersity evolved differently between these categories: most plasticized solvent-cast scaffolds narrowed from an initial PDI of about 1.9–2.3 to final values of 1.1–1.6, whereas nonplasticized samples instead maintained or slightly increased their PDI, rising from around 2.1 initially to 2.4–2.6 at the end of incubation (Figure S6C). This divergence likely reflects combined effects of heterogeneous chain scission, selective loss/dissolution of low-M_w_ fragments and local autocatalytic hydrolysis in more hydrophilic matrices.^35^ Across plasticized scaffolds the transition to a glassy state broadly coincided with M_w_ entering the ∼1–2 kDa window. At this stage, the samples were below their T_g_ at room temperature, which hindered detailed SEM inspection. For selected scaffolds examined at earlier degradation stages, however, collapse of pore walls and progressive coalescence were observable, with remnants of the original porous structure still discernible within the densifying matrix (Figure 4).

**Figure 4:**
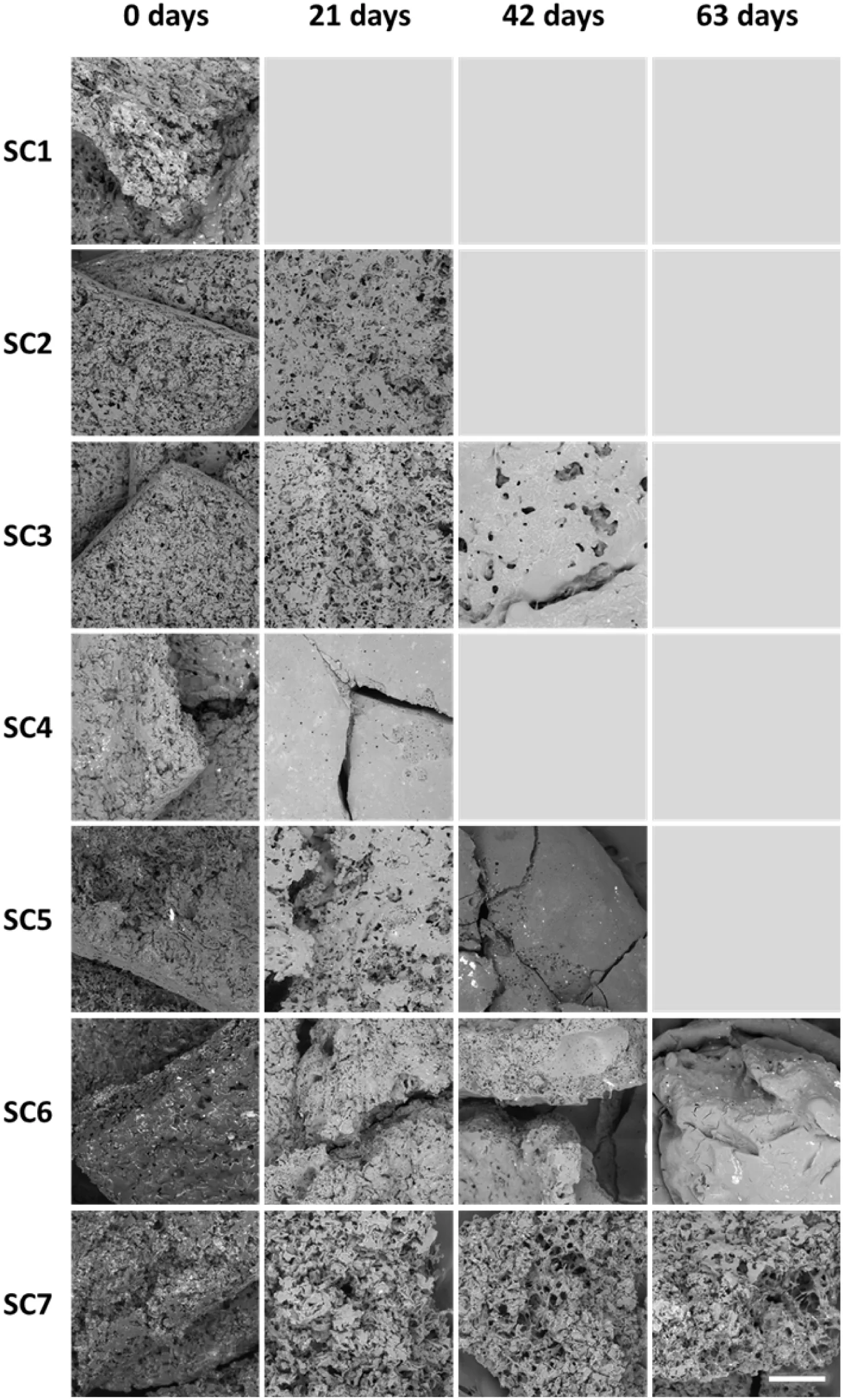
SEM images of solvent-cast Mup-PLGA scaffolds (SC1–SC7) at selected time points during in vitro degradation (0, 21, 42, and 63 days). Gray squares indicate samples in a glassy state, not suitable for SEM visualization. Scale bar: 500 μm.

SEM analysis therefore reflected the molecular trends observed during degradation. SC1 rapidly lost porosity and appeared glassy already at 21 days. SC2 and SC4 reached a glassy state by 42 days, while SC3 and SC5 did so by 63 days. SC6 did not enter a glassy state macroscopically, but SEM images revealed complete pore coalescence by day 63, leaving no discernible porosity. In contrast, the nonplasticized SC7 maintained an open porous architecture throughout the entire incubation period. The higher stability of SC7 compared to its plasticized counterparts highlights the role of plasticizers in lowering the softening point and promoting structural collapse. Overall, pore collapse, melting, and coalescence in the less stable scaffolds were most likely driven by the progressive decrease in T_g_ accompanying molecular-weight decline during degradation. Such behavior is consistent with previous reports linking loss of porosity to the accumulation of soluble oligomers during advanced chain scission.^36,37^ The ES1 and ES2 electrospun mats contracted macroscopically upon immersion in degradation medium but remained structurally stable thereafter, with degradation kinetics closely matching their nonplasticized, high-M_w_ solvent-cast analogue SC7 (Figure 5).

**Figure 5:**
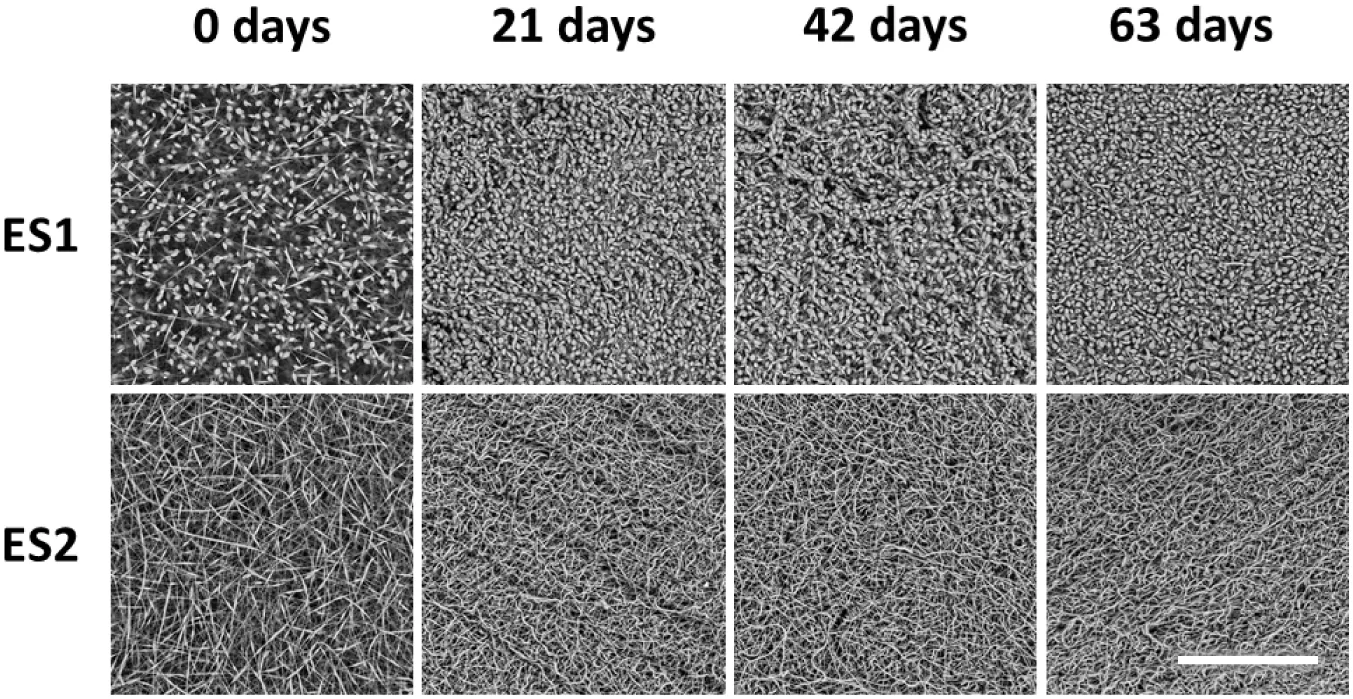
SEM images of electrospun Mup-PLGA mats (ES1 and ES2) at selected time points during in vitro degradation. Scale bar: 150 μm.

Scaffold degradation was therefore determined by copolymer ratio, molecular weight, and plasticizer content, while the fabrication method (solvent casting vs electrospinning) showed no apparent effect.

Chromatographic analysis of release media did not reveal discrete MUP peaks at any stage of the degradation study, irrespective of scaffold type (Figures S7, S8). This outcome indicates that ester bonds within the PLGA backbone were cleaved preferentially, while the covalent linkage to MUP remained more stable under the tested conditions. In nonplasticized scaffolds the chromatograms were largely featureless throughout, whereas in plasticized systems containing TEC, broad, nondiscernible peak clusters appeared and intensified over time. Their onset depended on polymer type: in the 50:50 series they were detectable from day 21 (SC1, SC2) or day 28 (SC3), while in the 75:25 series they emerged later – at day 49 for SC4, faintly at day 63 for SC5, and not at all for SC6, SC7 and electrospun ES1 and ES2 scaffolds (Figures S7, S8). These unresolved features are most consistent with complex mixtures of soluble degradation products, potentially including oligomeric conjugates of PLGA and MUP, although contributions from drug-free PLGA oligomers cannot be excluded. The appearance of these soluble species coincided with the late-stage molecular weight decline observed in GPC, consistent with earlier reports that short-chain PLGA oligomers become water-soluble once chain scission proceeds beyond a critical threshold.^38^ In all TEC-containing samples, a distinct TEC peak was detectable at the start of the experiment, with variations in retention time observed during degradation, likely reflecting pH changes in the release medium. Overall, these results suggest that MUP was not released as a free molecule but remained associated with the degrading polymer matrix throughout the study.

### Antimicrobial Activity of Mup-PLGA Biomaterials

Antibacterial activity of the Mup-PLGA scaffolds was evaluated using both diffusion-based and contact-based assays. In the disk diffusion assay, inhibition zones were observed only for electrospun samples (ES1, ES2), whereas none of the solvent-cast scaffolds (SC1–SC7) produced measurable halos (Figure 6B). This indicates that only electrospun materials released diffusible species capable of inhibiting bacterial growth under these conditions, although the zones remained relatively small.

**Figure 6:**
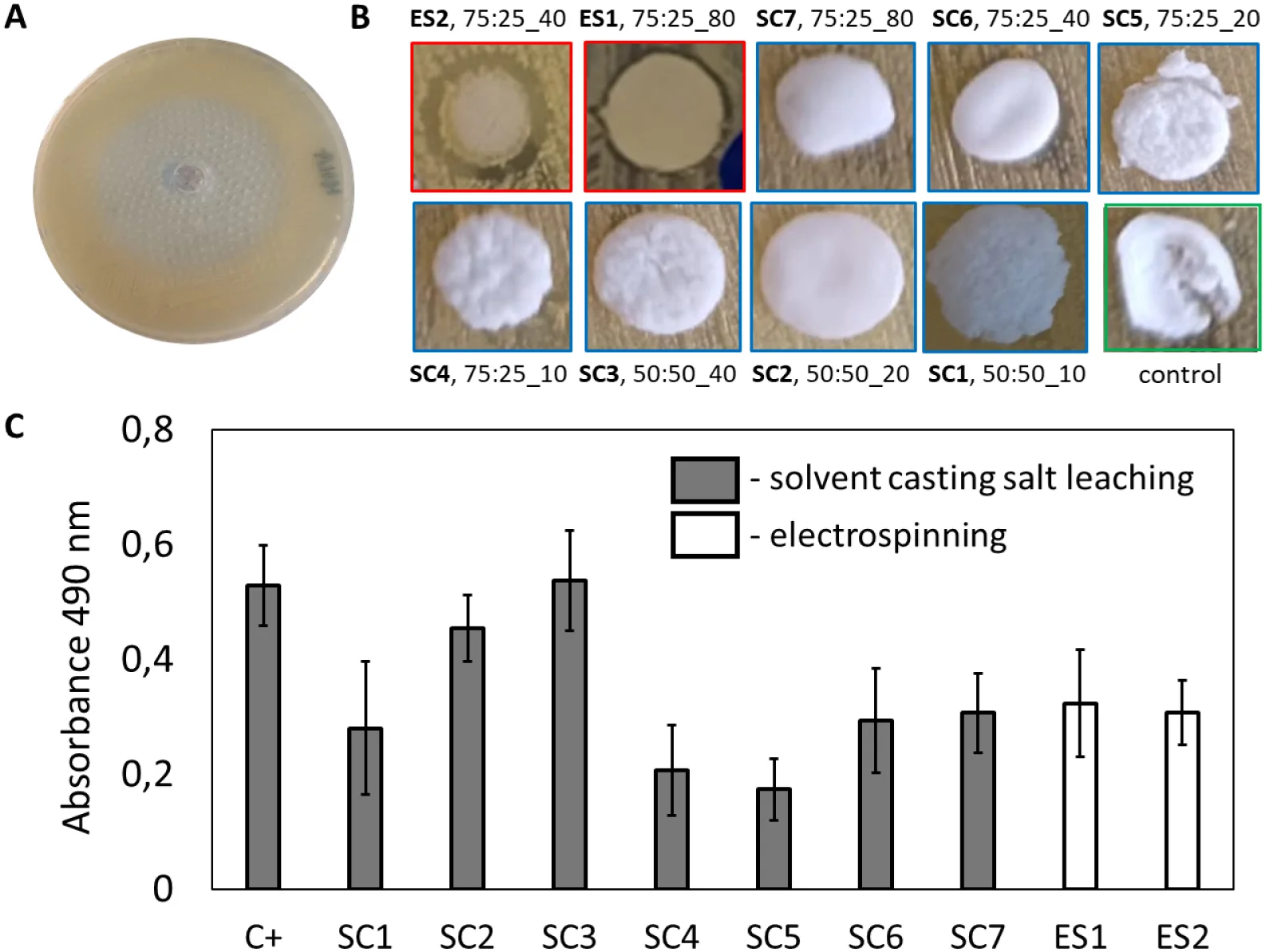
(A) Inhibition zone formed by the positive control (commercial MUP ointment) against *Staphylococcus aureus* ATCC 6538. (B) Representative images of inhibition zones for scaffold samples: solvent-cast (SC1–SC7, blue frames) and electrospun (ES1–ES2, red frames), with corresponding conjugate variants indicated; negative control shown in green. (C) Antibacterial activity under direct-contact conditions assessed by a TTC-based assay. Absorbance at 490 nm reflects metabolic activity of adherent *S. aureus* after 3 h of contact. Error bars represent standard deviation.

Differences between scaffold types may be influenced by structural evolution during incubation. In degradation studies, plasticized solvent-cast scaffolds underwent progressive pore collapse and densification, which likely reduced effective surface area and restricted interaction with the surrounding medium. By contrast, electrospun mats retained a high surface-to-volume ratio, which may facilitate interfacial interactions and limited outward transport of low-molecular-weight species, making small inhibition zones detectable only for ES samples. This interpretation is also consistent with broader electrospinning literature, where high porosity and specific surface area are repeatedly invoked to explain enhanced antimicrobial performance in loaded mats.^39^

In contrast, the TTC-based contact assay revealed differences in metabolic activity of *Staphylococcus aureus* across the tested scaffolds (Figure 6C). The strongest reduction was observed for low-molecular-weight conjugates, whereas samples derived from higher-molecular-weight polymers showed higher residual metabolic activity, approaching control values. This trend reflects the decreasing fraction of MUP incorporated per polymer chain with increasing molecular weight, as expected for drug-initiated polymerization. For comparable conjugates, 75:25_40 and 75:25_80, electrospun and solvent-cast scaffolds exhibited similar activity, indicating that under direct-contact conditions the antibacterial effect is governed mainly by conjugate composition rather than scaffold format.

To further evaluate the antibacterial potential of electrospun scaffolds, release media collected after 1–3 weeks of incubation were tested using a broth microdilution assay. No inhibition of *S. aureus* growth was observed for any of the collected media. Under the same conditions, the MIC of MUP was approximately 0.125 μg/mL. The absence of antibacterial activity in the release media relative to this MIC suggests that any released species, if present, remained below therapeutically relevant levels. This finding is consistent with the degradation study, in which no discrete MUP peaks were detected by HPLC in release samples. Instead, only selected plasticized solvent-cast scaffolds showed broad, unresolved chromatographic features at later time points, attributed to soluble low-molecular-weight degradation products, potentially including oligomeric Mup-PLGA species, whereas such features were not observed for ES1 and ES2. The differences between the agar diffusion and release-medium assays can be attributed to the distinct mass-transport conditions. In the disk diffusion assay, hydration of the electrospun mats at the agar interface may induce superficial release of a small bioactive fraction from the highly accessible fibrous surface, producing an inhibitory concentration only within a narrow region surrounding the sample. The small halos observed for ES1 and ES2 therefore indicate limited short-range diffusion. The released fraction may comprise trace surface-accessible MUP or low-molecular-weight MUP-containing species; however, its chemical identity could not be established because no discrete free-MUP peak was detected by HPLC. In the release-medium assay, any diffusible material was dispersed throughout the bulk medium and did not reach an antibacterial concentration, as confirmed by the absence of growth inhibition after 1–3 weeks of incubation. The lack of inhibition zones around SC1–SC7 is consistent with the lower surface accessibility and more restricted transport within the solvent-cast matrices. Together with the direct-contact results, these findings indicate that the scaffolds exhibit predominantly surface-mediated antibacterial activity, with only limited local diffusion from the electrospun mats. This behavior contrasts with classical loading-based electrospun systems, where antibacterial efficacy is primarily governed by drug release into the surrounding medium. Mupirocin-loaded PLLA scaffolds have been shown to maintain drug concentrations above MIC levels over extended periods, resulting in large inhibition zones and sustained antibacterial activity.^40^ Similarly, PCL/chitosan double-layer nanofibrous dressings exhibit an initial burst release followed by prolonged release over several days, which correlates with strong activity against *S. aureus*, *E. coli*, and *P. aeruginosa*.^41^ More broadly, in many electrospun wound dressing systems based on physical drug incorporation, high cumulative release-often reaching 70–90% within hours to days-is directly associated with pronounced inhibition zones and effective bacterial killing, reinforcing the central role of diffusion-driven mechanisms in these materials.^42^

Against this background, Mup-PLGA based scaffolds appear fundamentally different. Under direct-contact conditions, several scaffolds reduced bacterial metabolic activity despite the lack of measurable antibacterial effect in release media. A similar distinction between released and contact-active fractions has been reported for PCL-based electrospun dressings loaded with thymol or carvacrol. In that system, the mats retained antibacterial efficacy, whereas exudates collected from the mats showed no antimicrobial activity, leading the authors to conclude that direct contact between bacteria and the loaded dressing was required for full bactericidal action.^43,44^

Overall, the results indicate that antibacterial activity of the Mup-PLGA scaffolds is predominantly surface-mediated, with only a limited contribution from diffusible species. In contrast to conventional drug-loaded wound dressings, where antimicrobial efficacy is largely governed by rapid or sustained release into the surrounding medium, the present conjugate system appears to retain activity primarily at the material–bacteria interface. Such contact-based behavior may be advantageous in limiting unnecessary exposure of bacteria to subinhibitory concentrations of antibiotics, which are known to promote the development of resistance. In this context, restricting antimicrobial action to the immediate vicinity of the scaffold surface could reduce selective pressure in the surrounding environment while maintaining local efficacy.

### In Vitro Cytocompatibility of Mup-PLGA Scaffolds

The effect of scaffold extracts on HaCaT metabolic activity was evaluated using the MTT assay (Figure 7).

**Figure 7:**
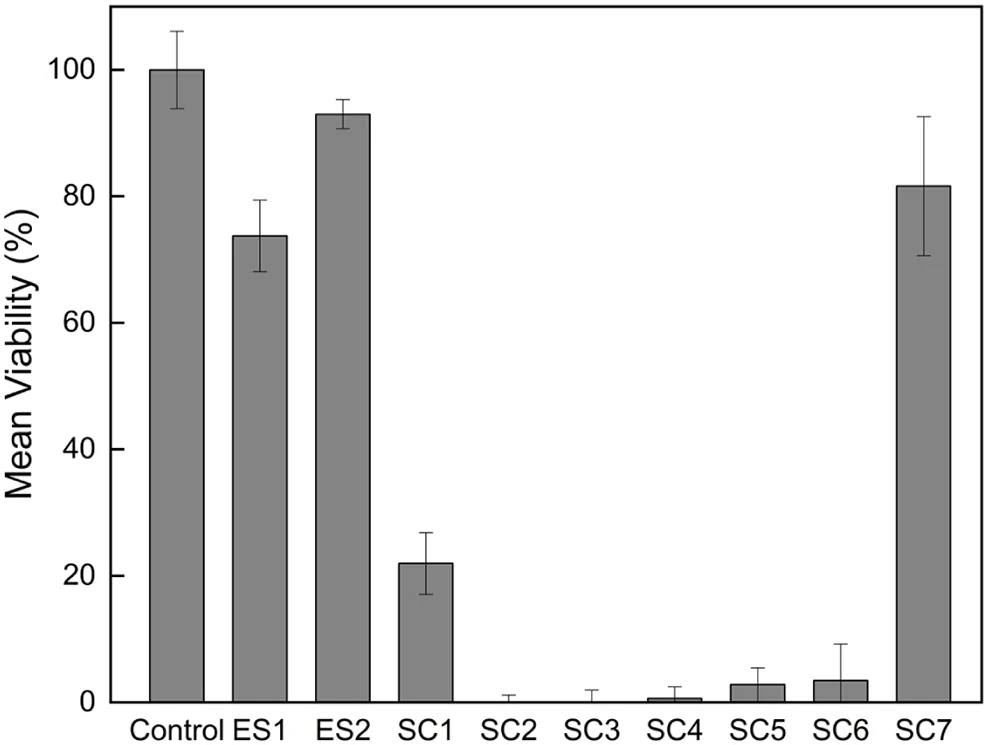
Viability of HaCaT cells following exposure to Mup-PLGA scaffold extracts, determined using the MTT assay. Values are expressed relative to untreated cells and presented as mean ± SD.

Both electrospun scaffolds maintained cell viability above the 70% cytotoxicity threshold, with values of 73.8 ± 5.6% for ES1 and 93.0 ± 2.3% for ES2. Among the solvent-cast scaffolds, SC7 also showed a noncytotoxic response, maintaining a viability of 81.6 ± 11.0%. In contrast, all TEC-containing solvent-cast formulations produced a marked reduction in metabolic activity. Cell viability was 22.0 ± 4.9% for SC1 and remained close to zero for SC2–SC6. The results indicate a clear difference between SC7, prepared from the 75:25_80 conjugate without TEC, and the TEC-containing solvent-cast formulations SC1–SC6. This pattern suggests that the cytotoxic response may be associated with TEC or with extractable components generated or retained during preparation of the plasticized scaffolds. The comparison between ES1 and SC6 is particularly relevant, as both were prepared from the 75:25_40 conjugate, but their extracts resulted in cell viabilities of 73.8% and 3.5%, respectively. In contrast, both ES2 and the corresponding solvent-cast SC7 formulation based on the 75:25_80 conjugate remained above the cytotoxicity threshold. Because all Mup-PLGA conjugates were synthesized using the same Sn­(Oct)_2_ conditions, whereas ES1, ES2, and SC7 remained above the 70% viability criterion, residual catalyst is unlikely to be the primary determinant of the observed formulation-dependent differences.

The consistent reduction in viability for all TEC-containing formulations indicates that the cytotoxic response was primarily associated with the extractable fraction of the plasticized matrices. TEC is water-soluble and has been shown to leach from PLGA films during exposure to aqueous media.^29^ Released TEC is therefore the most plausible primary contributor to the observed effect. However, TEC may also modify the behavior of the polymer matrix: the incorporation of 7% TEC accelerated the degradation of implanted PLGA membranes,^45^ while rapid PLGA degradation and the resulting acidic environment have been associated with reduced cell viability.^46^ The response observed for SC1–SC6 may therefore result from the combined presence of TEC and low-molecular-weight PLGA-derived products in the extracts. Importantly, the progressive tissue integration reported for TEC-containing PLGA membranes indicates that its biological effect is formulation-dependent rather than indicative of the inherent incompatibility of TEC.^45^ Therefore, optimization of the TEC concentration is required to limit the release of cytotoxic extractables while maintaining the desired processing and mechanical properties.

## Conclusions

This work demonstrates that drug-initiated ROP provides an alternative strategy for incorporating MUP into a biodegradable polyester matrix. In contrast to conventional loading approaches, where the drug is physically entrapped and may suffer from inhomogeneous distribution, burst release, or leaching, covalent incorporation ensures uniform distribution of the drug along the polymer chains, eliminates issues related to phase separation during processing, and prevents uncontrolled drug loss from the material. At the same time, the resulting conjugates retain sufficient processability to be fabricated into scaffolds with different architectures.

The results further show that successful processing of such conjugates into functional biomaterials is governed by a combination of factors, among which solubility plays a critical role. These constraints are system-specific and must be considered individually for each polymer–drug system, as they directly determine accessible fabrication routes and ultimately the structure of the resulting materials. Within the processable range, both solvent-cast and electrospun scaffolds were obtained, indicating that intrinsic material properties primarily govern degradation behavior.

Combined physicochemical and microbiological analyses indicate that these materials do not behave as classical release systems. Despite progressive degradation, MUP remained associated with the polymer matrix, and no therapeutically relevant concentrations of free drug were detected in the surrounding medium. Antibacterial activity was observed predominantly under direct-contact conditions and correlated with conjugate composition, reflecting the inherent relationship between molecular weight and MUP content in drug-conjugated systems. This behavior places Mup-PLGA scaffolds between conventional release-based dressings and nonleaching antimicrobial materials. The absence of bulk release, together with retained antibacterial activity at the material interface, suggests a localized mode of action. Such a feature may reduce the exposure of bacteria to subinhibitory antibiotic concentrations in the surrounding environment, potentially limiting the development of resistance while maintaining efficacy at the material surface. At the same time, it was shown that scaffold architecture influenced the mode of antibacterial activity. This underscores that both molecular design and structural organization must be considered together when developing conjugate-based antimicrobial biomaterials. Extract-based MTT testing showed formulation-dependent cytocompatibility. Mean cell viability remained above the 70% criterion for ES1, ES2, and the nonplasticized SC7 scaffold, whereas all TEC-containing solvent-cast scaffolds were cytotoxic, indicating that these formulations require further optimization before biological application.

## Supplementary Material



## Notes

Apparent recovery of mupirocin during catalyst-free incubation in the LA/GA melt; DSC thermograms of Mup-PLGA conjugates; SEM micrographs from electrospinning trials; GPC analysis during in vitro scaffold degradation; HPLC chromatograms of release media (PDF)
