# Supplementary material for: Drug-Initiated Synthesis and Processing of Mupirocin–PLGA Conjugates for Biodegradable Antibacterial Scaffolds
Source: ACS Omega. 2026 Oct 5;11(41):63299–311. doi: 10.1021/acsomega.6c06646 (PMC13688565; doi:10.1021/acsomega.6c06646)
Supplement: Supplementary file 1 [file ao6c06646_si_001.pdf]

Drug-initiated synthesis and processing of mupirocin–PLGA conjugates for biodegradable antibacterial scaffolds.

Tomasz Urbaniak[a], Zuzanna Podgórniak[a], Adam Junka[b, c], Jolanta Harasiuk[d], Witold Musiał[a]*

[a] Department of Physical Chemistry and Biophysics, Faculty of Pharmacy, Wroclaw Medical University, Borowska 211, 50-556 Wrocław, Poland

[b] Platform for Unique Model Applications (PUMA), Division of Translational Technologies, Faculty of Pharmacy, Wroclaw Medical University, Borowska 211, 50-556 Wrocław, Poland

[c] College of Life Sciences and Medicine, Zhejiang Sci-Tech University, 928 Second Avenue, Xiasha Higher Education Zone, Hangzhou, Zhejiang 310018, P.R. China

[d] Department of Molecular and Cellular Biology, Faculty of Pharmacy, Wroclaw Medical University, Borowska 211a, Wroclaw 50-556, Poland

*Corresponding author; e-mail address: witold.musial@umw.edu.pl (W. Musial)

Supporting Information

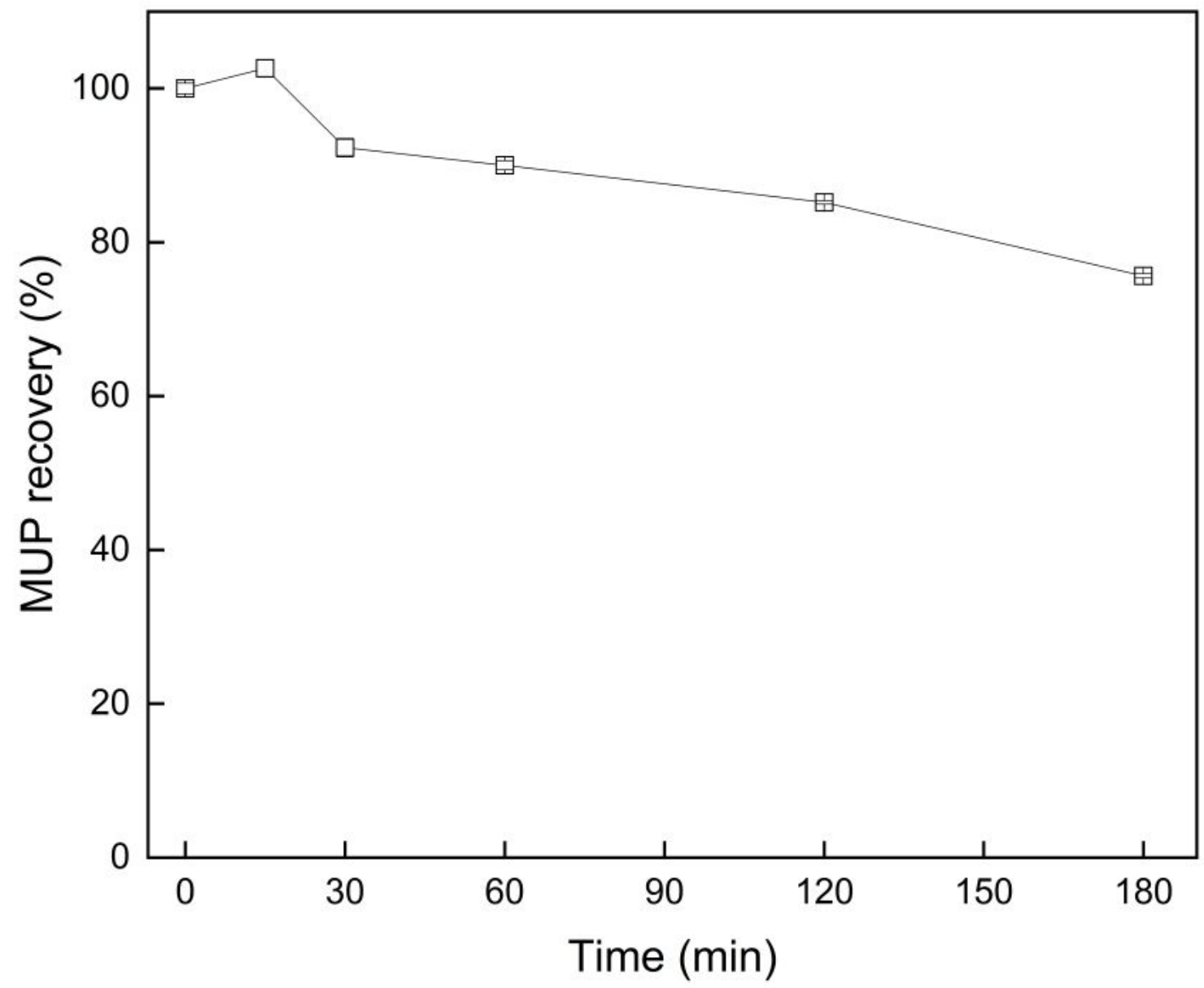

Figure S1. Apparent recovery of intact mupirocin during incubation in the catalyst-free LA/GA melt at 130 ˚C. Values were normalized to 0 min; error bars indicate the SD of repeated HPLC injections.

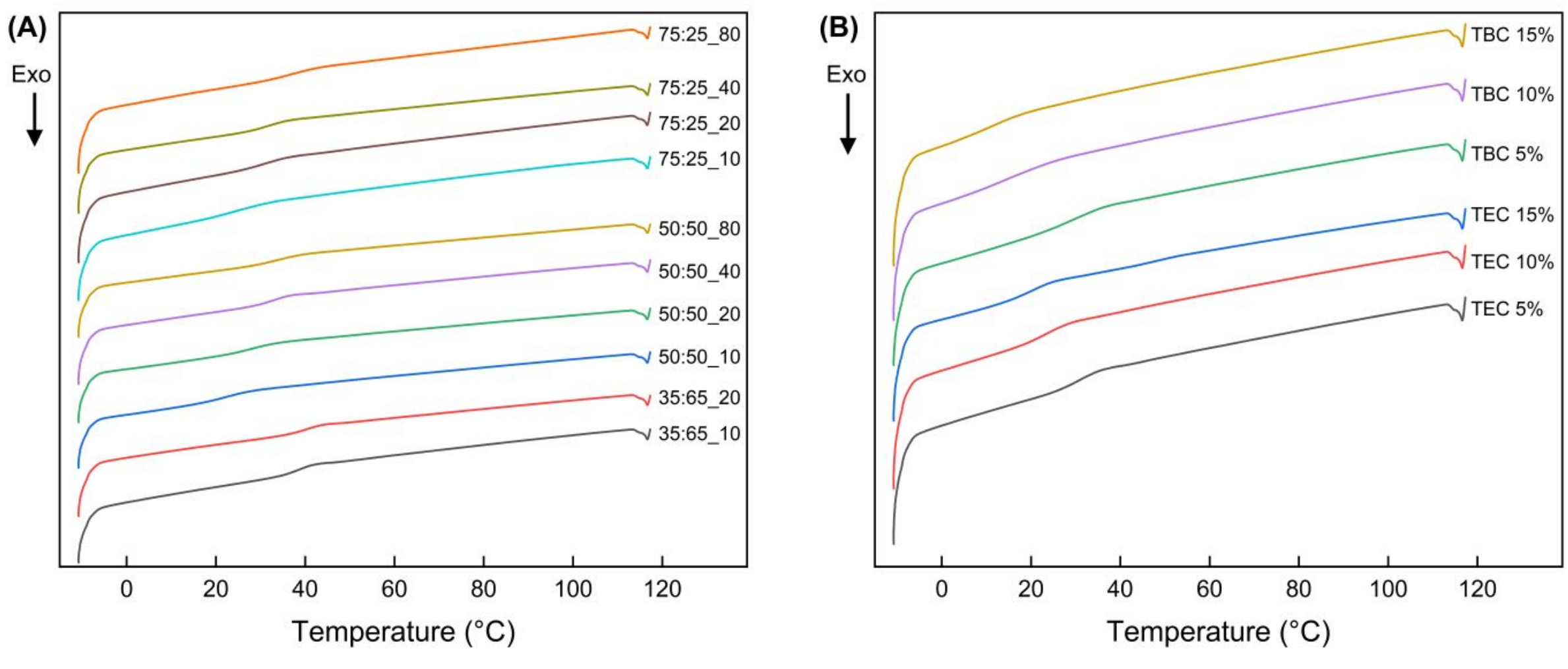

Figure S2. Differential scanning calorimetry (DSC) thermograms of Mup-PLGA conjugates. (A) Representative thermograms of all synthesized products with varying LA:GA ratios and

molecular weights. (B) Thermograms of the 75:25_10 Mup-PLGA conjugate formulated with different concentrations of triethyl citrate (TEC) and tributyl citrate (TBC).

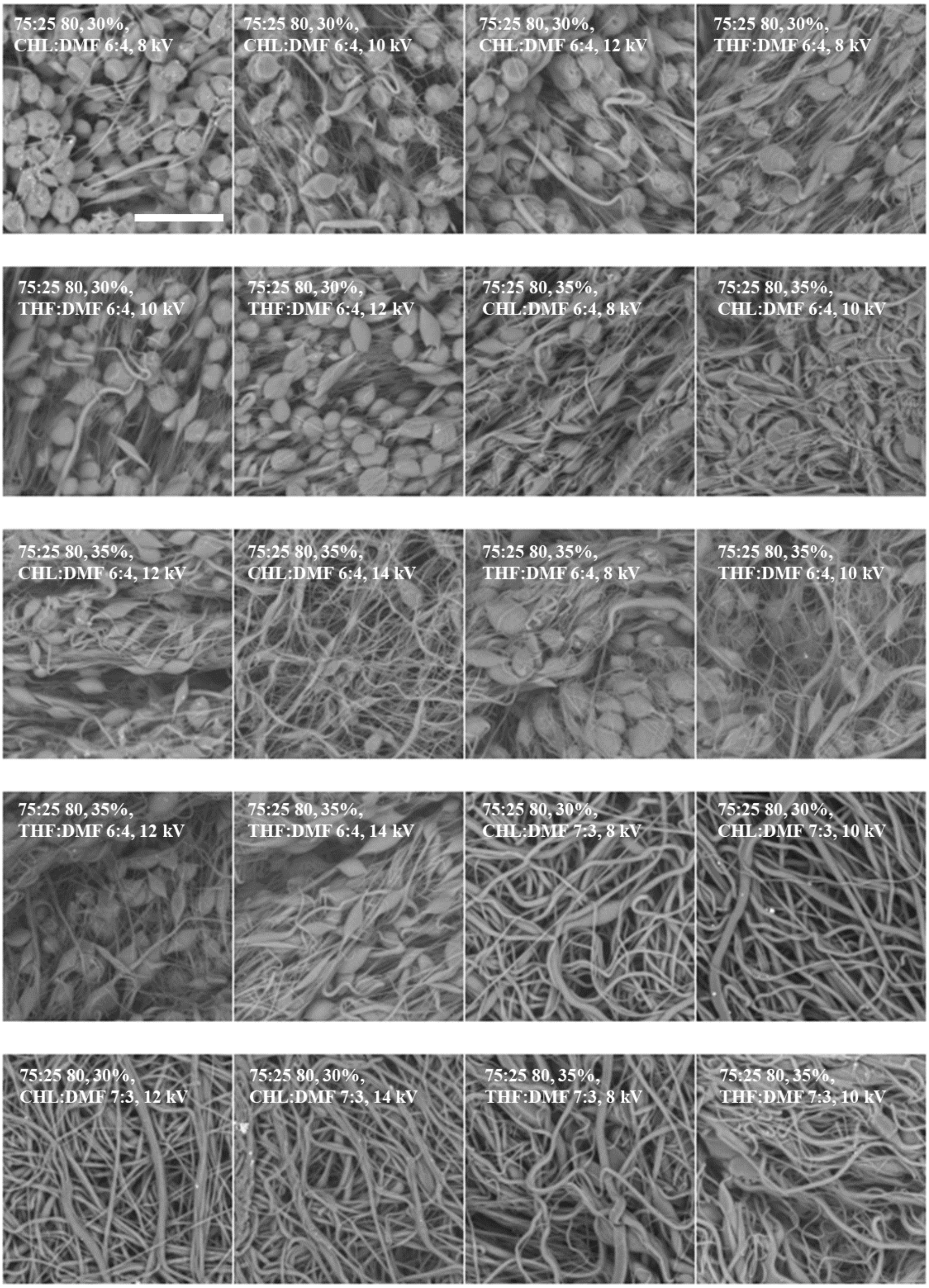
75:25 80, 30%, CHL:DMF 6:4, 8 kV
75:25 80, 30%, CHL:DMF 6:4, 10 kV
75:25 80, 30%, CHL:DMF 6:4, 12 kV
75:25 80, 30%, THF:DMF 6:4, 8 kV
75:25 80, 30%, THF:DMF 6:4, 10 kV
75:25 80, 30%, THF:DMF 6:4, 12 kV
75:25 80, 35%, CHL:DMF 6:4, 8 kV
75:25 80, 35%, CHL:DMF 6:4, 10 kV
75:25 80, 35%, CHL:DMF 6:4, 12 kV
75:25 80, 35%, CHL:DMF 6:4, 14 kV
75:25 80, 35%, THF:DMF 6:4, 8 kV
75:25 80, 35%, THF:DMF 6:4, 10 kV
75:25 80, 35%, THF:DMF 6:4, 12 kV
75:25 80, 35%, THF:DMF 6:4, 14 kV
75:25 80, 30%, CHL:DMF 7:3, 8 kV
75:25 80, 30%, CHL:DMF 7:3, 10 kV
75:25 80, 30%, CHL:DMF 7:3, 12 kV
75:25 80, 30%, CHL:DMF 7:3, 14 kV
75:25 80, 35%, THF:DMF 7:3, 8 kV
75:25 80, 35%, THF:DMF 7:3, 10 kV

Figure S3. Scanning electron microscopy (SEM) micrographs of Mup-PLGA electrospinning trials conducted under varying processing conditions (indicated on each image). All images were acquired at the same magnification; scale bar = 20 μm.

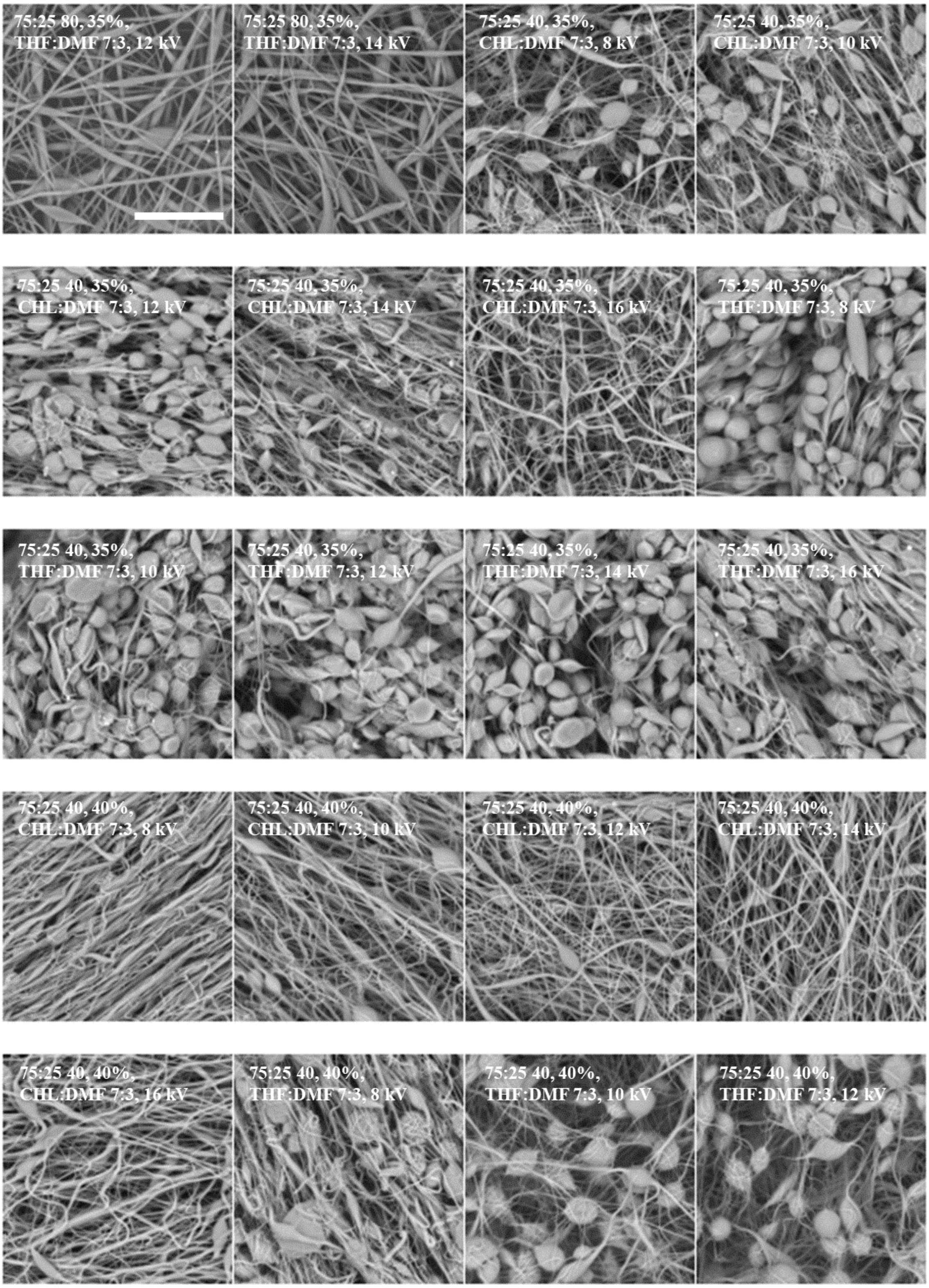

Figure S4. Scanning electron microscopy (SEM) micrographs of Mup-PLGA electrospinning trials conducted under varying processing conditions (indicated on each image). All images were acquired at the same magnification; scale bar = 20 μm.

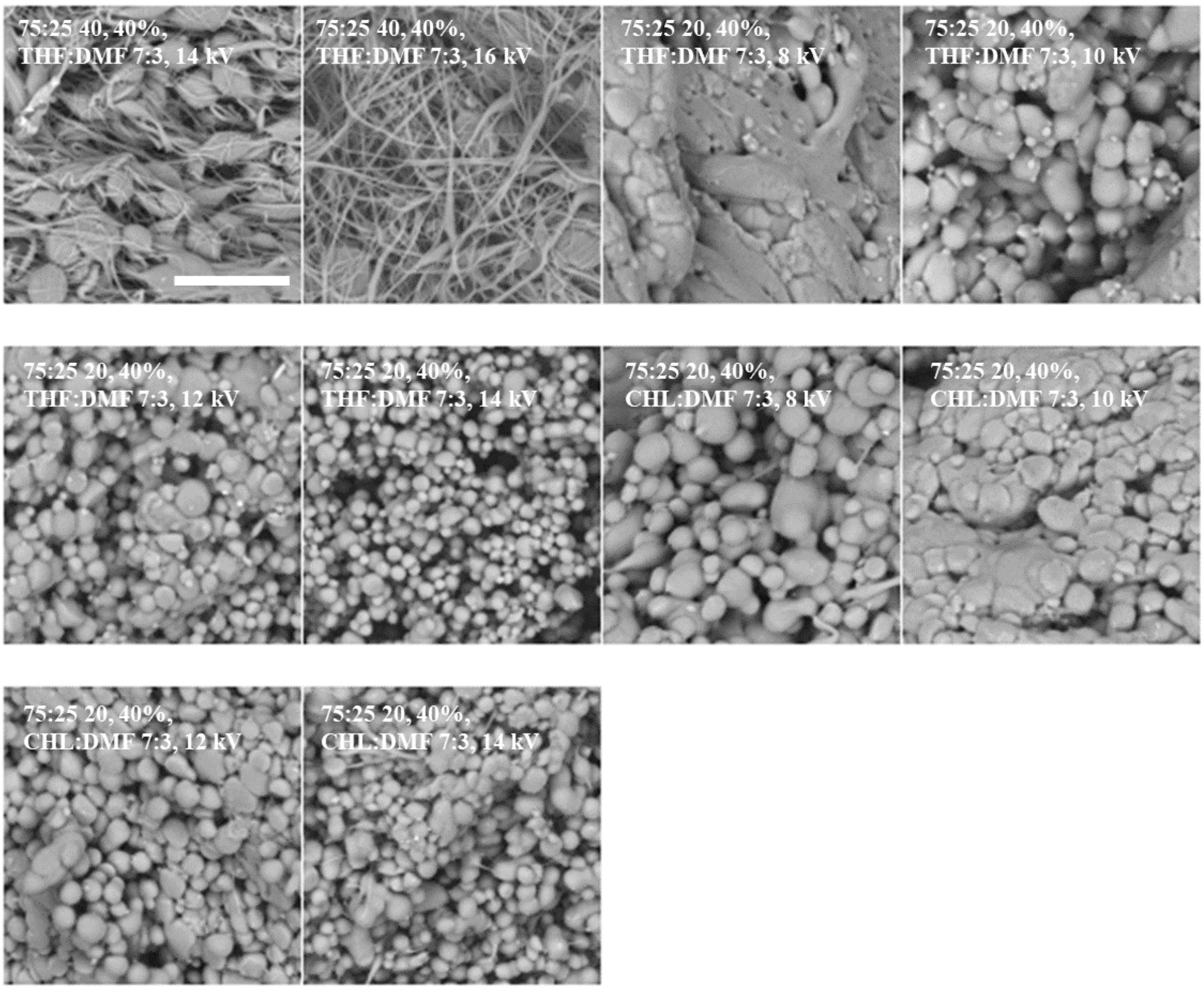

Figure S5. Scanning electron microscopy (SEM) micrographs of Mup-PLGA electrospinning trials conducted under varying processing conditions (indicated on each image). All images were acquired at the same magnification; scale bar = 20 μm.

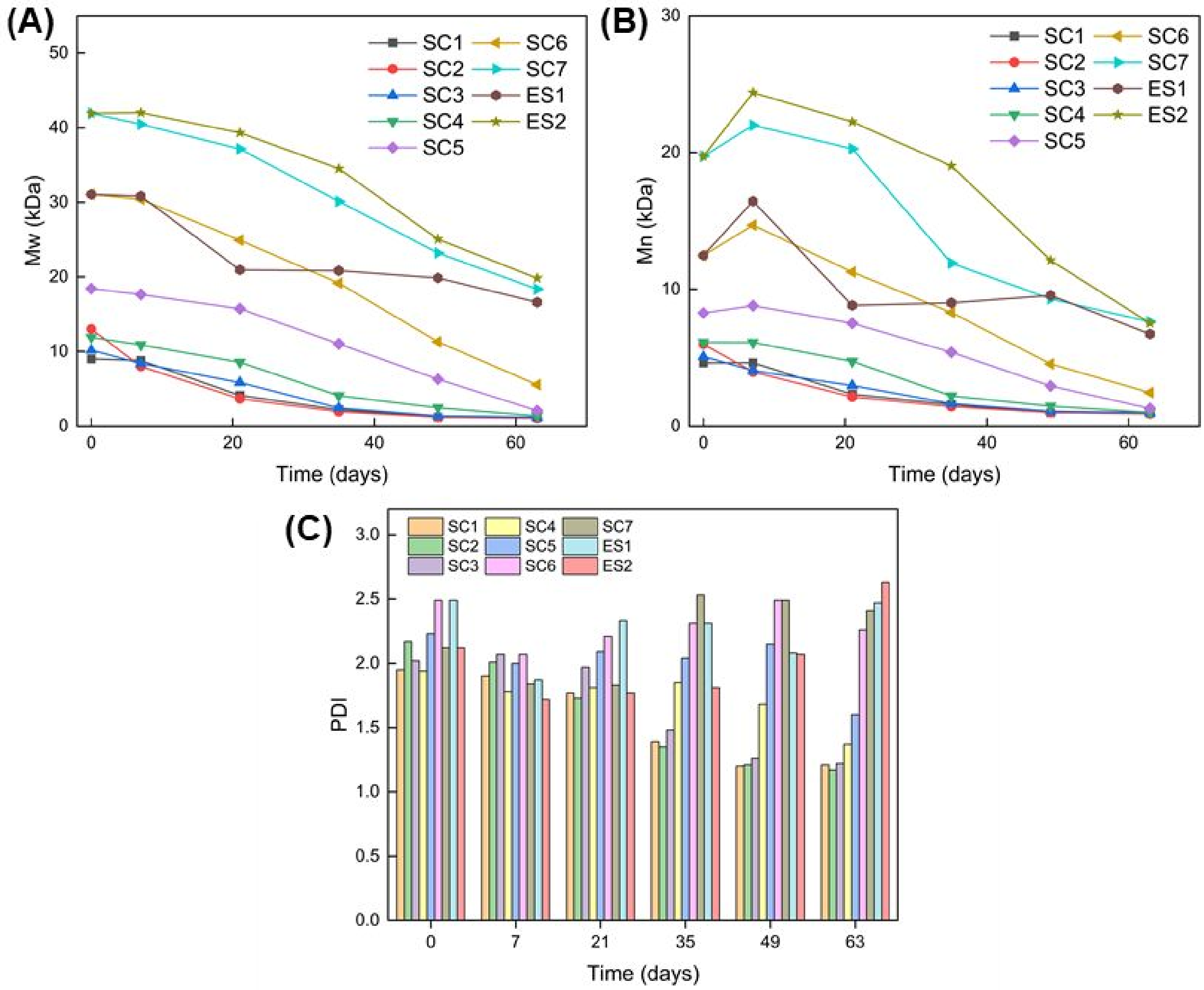

Figure S6. Gel permeation chromatography (GPC) analysis of Mup-PLGA conjugates during 63-day in vitro degradation. (A) Changes in weight-average molecular weight (Mw). (B) Changes in number-average molecular weight (Mn). (C) Evolution of polydispersity indices (PDI). Data are shown for seven solvent-cast samples (SC1–SC7) and two electrospun samples (ES1 and ES2).

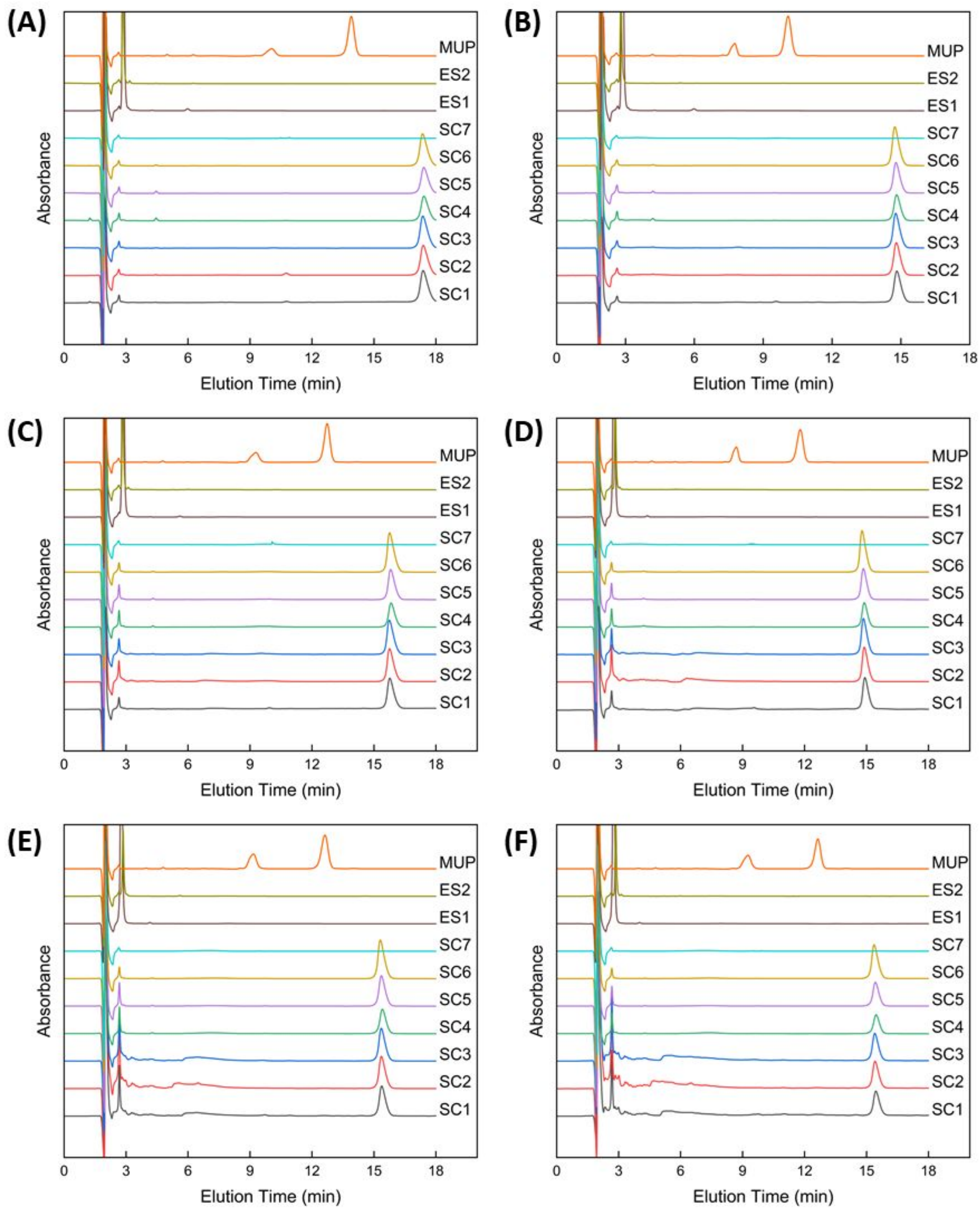

Figure S7. Stacked HPLC chromatograms obtained during in vitro degradation experiments showing mupirocin release from all scaffold variants (SC1–SC7 and ES1–ES2) at selected time points: day 3 (A), 7 (B), 14 (C), 21 (D), 28 (E), and 35 (F). Each panel includes the reference chromatogram of pure mupirocin (MUP).

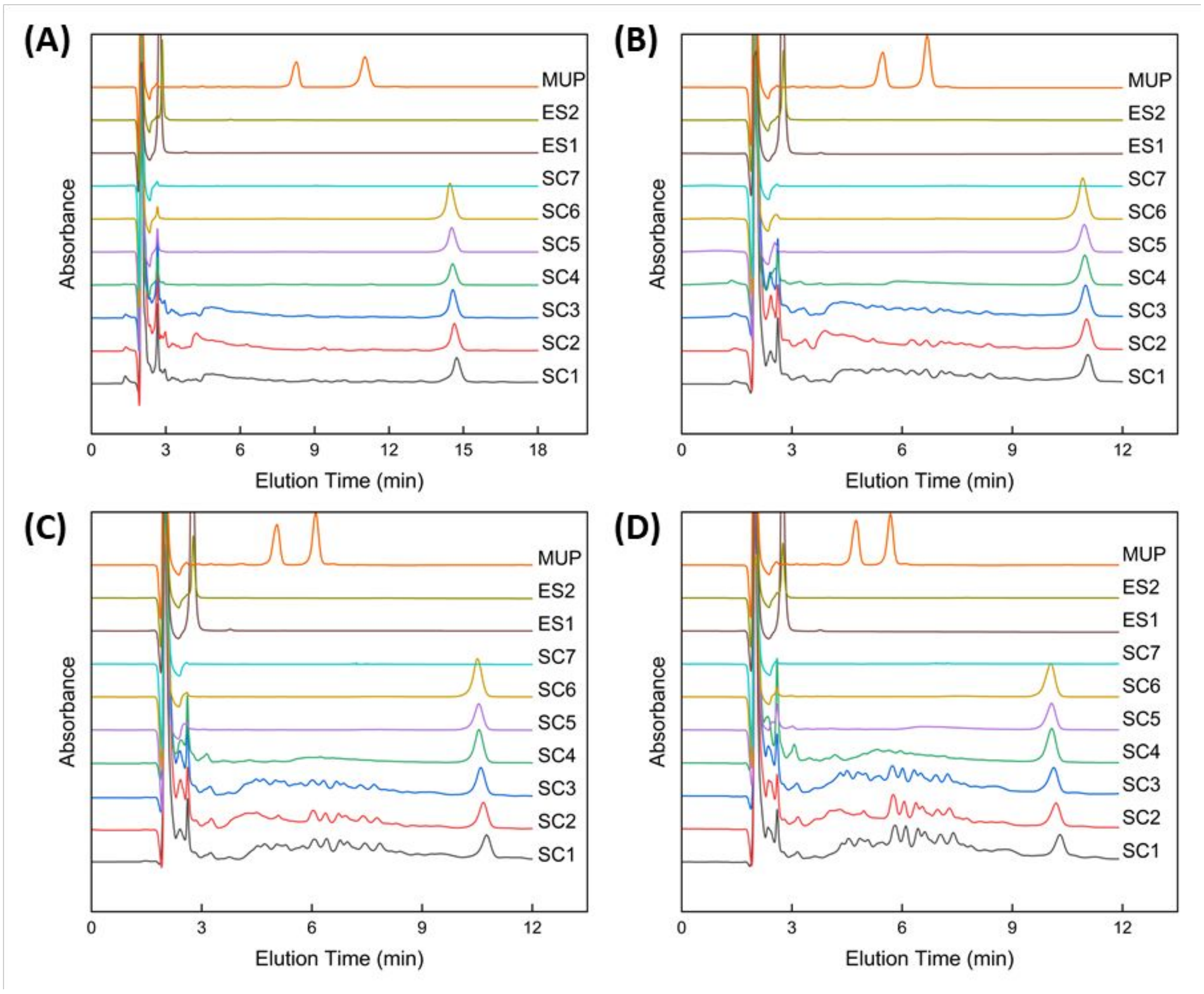

Figure S8. Stacked HPLC chromatograms obtained during in vitro degradation experiments showing mupirocin release from all scaffold variants (SC1–SC7 and ES1–ES2) at selected time points: day 42 (A), 49 (B), 56 (C), and 63 (D). Each panel includes the reference chromatogram of pure mupirocin (MUP).
